# Associations between maternal older age, family environment and parent and child wellbeing in families using assisted reproductive techniques to conceive

**DOI:** 10.1016/j.socscimed.2009.02.036

**Published:** 2009-06

**Authors:** J. Boivin, Frances Rice, Dale Hay, Gordon Harold, Allyson Lewis, Marianne M.B. van den Bree, Anita Thapar

**Affiliations:** a School of Psychology, Cardiff University, Park Place, Cardiff, CF10 3AT, Wales, United Kingdom; b Department of Psychology, University College London, London, United Kingdom; c Department of Psychological Medicine, School of Medicine, Cardiff University, Cardiff, Wales, United Kingdom

**Keywords:** In vitro fertilisation (IVF), Middle childhood, Age, Assisted conception, Older mother, Family environment, Gamete donation, UK, Delayed parenting

## Abstract

Maternal age effects on parenting and family outcomes are of increasing interest because of the demographic shift toward older maternal age at first birth. Maternal age is also of interest because of the greater use of assisted reproductive techniques (ART) to bypass age-related infertility in couples trying to conceive late in the reproductive life cycle of the woman. The aim of the present study was to investigate maternal age effects associated with delayed parenting by comparing families of mothers who gave birth at a younger (<31 years) or older (>38 years) age and to ascertain whether associations were linear associations by comparing these groups to women who had conceived in between these ages (i.e., >31 and <38 years). All children (4–11 year olds) were first-born and conceived using ART. Participants were recruited from one of 20 fertility clinics and mothers (*n* = 642) and fathers (*n* = 439) completed a postal questionnaire about demographic and reproductive characteristics, family environment as well as parent and child wellbeing. Our results demonstrate that parenthood via assisted conception later in the reproductive life cycle is not associated with a negative impact on child wellbeing. Despite maternal age-group differences on demographic (education, income) and reproductive characteristics (bleeding during pregnancy, caesarean rate, breast feeding), and parental warmth and depressive symptoms, child wellbeing was similar across mother age groups. We conclude that the parenting context is different for older mother families (more depressive symptoms in mothers and fathers, less expressed warmth in the couple) but that this difference is not associated with child wellbeing in early and middle childhood.

## Introduction

The demography of parenting is changing and nowhere is this more evident than in the timing of parenthood. Average maternal age at first birth in 1968 was about 23 years whereas it is now between 30 and 31 years of age (Office of National Statistics, United Kingdom (UK) (ONS, 2007), Table 1.6). In 1963 the number of first births per 1000 women in the United States was 7.2 and 2.6 for age groups 30–34 and 35–39 (respectively) and this number rose to 28.6 and 10.3 in 2003 (Centers for Disease Control and Prevention, Tables 1,2, accessed 22.12.08). Given this demographic shift, maternal age effects on parenting and family outcomes have become of interest. However, existing studies focus on a restricted time in infancy and a narrow range of parent and child outcomes and generally fail to take into account confounding effects of a history of fertility difficulties or marital/partnership variables (e.g., longevity, expressed warmth). The aim of the present study was to ascertain maternal age effects associated with delayed parenting by comparing families of mothers who gave birth at a younger (<31 years) or older (>38 years) age on family environment and parent and child wellbeing. All children (4–11 years of age) were first-born and conceived with assisted reproductive techniques (ART).

There is inconsistency in the age that defines an older mother and this problem makes it difficult to synthesize research bearing on age and parenting. Definitions vary according to the decade in which studies were published, the target age group and the specific outcome investigated. For example, in two studies carried out in 1987 and 1997 (Berryman & Windridge, 1997; Robinson, Garner, Gare, & Crawford, 1987) mothers giving birth at 35 years were categorised as older mothers whereas the same age defined the younger mothers in a recent 2007 investigation (McMahon, Gibson, Allen, & Saunders, 2007). In research targeting teenage mothers the non-teenage mother is often classified as ‘older’ when in reality she may be having her child at a normative age for that population (Waters, 2004). Medical studies often use age >34 years to define older mothers because this age is associated with changes in obstetrical practice (e.g., increase in prenatal screening) but even this cut-off varies depending on the specific outcome (Collins & Crosignani, 2005). In light of these inconsistencies between literatures we restricted our survey of age and parenting research to results of studies where the older mother was the primary focus of the research and where her psychosocial data were compared to a younger cohort of more or less on-time mothers who were considered to have given birth at the typical or normative age for first births in that population. Surveys on families using assisted conception where mothers tend to be older (e.g., Barnes et al., 2004) were not reviewed unless they met these criteria.

Results of studies comparing younger (on-time) and older mothers on pregnancy and parenting variables essentially show positive outcomes in older mothers. Although older mothers show less attachment to their foetus in mid-pregnancy (Berryman & Windridge, 1996) attachment is comparable in late pregnancy (McMahon et al., 2007). Observational data show that older mothers provide more sensitivity (warmth, emotional connection) and structure (provision of structured environment) to five-month old infants (Bornstein, Putnick, Suwalsky, & Gini, 2006) and richer cognitive experiences for one year olds (Berryman, Thorpe, & Windridge, 1995) than younger mothers, even after controlling for socio-economic factors (Bornstein et al., 2006). Whether this relative advantage continues in later childhood or translates into better child outcomes needs more research attention because maximum infant age in older mother cohort studies is one year (Berryman & Windridge, 1997) and child outcomes have not yet been examined in older mother families.

The better parenting practices in older mother families have been attributed to the so-called maternal maturity hypothesis (Hofferth, 1987), that is, to the fact that older mothers have accrued life experiences, wisdom, financial and social resources, and a more varied coping repertoire that promotes a more responsive family environment (Bornstein et al., 2006). However, the maternal age effects observed thus far could also be due to other factors associated with reproducing later in the female reproductive life cycle: a history of infertility, pre-existing educational and personality differences. A significant proportion of older mothers report experiencing reproductive difficulties (e.g., Bornstein et al., 2006; Robinson et al., 1987) or longer time to conceive (McMahon et al., 2007; Robinson et al., 1987) and, because of their age, are at an increased risk for miscarriage and a variety of gestational and labour complications (Bewley, Davies, & Braude, 2005). Furthermore, older mothers are more likely to need the donated oocytes of a younger woman to conceive due to age-related decline in the quality of their own oocytes that makes them unlikely to fertilise (Collins & Crosignani, 2005). As a result, previously infertile women worry more about their pregnancy (e.g., Leiblum, Kemmann, & Taska, 1990) and such concerns could explain the lower engagement of older mothers in the early stages of pregnancy (Berryman & Windridge, 1996; Robinson et al., 1987). Indeed, in the only investigation controlling for infertility history, attachment was similar in younger and older infertile mothers (McMahon et al., 2007). Follow-up studies with children conceived with fertility treatment show that previously infertile mothers as compared to their never infertile counterparts show less parenting stress (Abbey, Andrews, & Halman, 1994) and tend to be warmer and more emotionally involved with their children (Golombok et al., 1996). Thus the disengagement in pregnancy but greater warmth and involvement in infancy observed in older parents could be due more to secondary effects of a so-called ‘hard-to-achieve pregnancy’ (Van Balen, 1998) than to the greater maturity of older mothers.

In studies comparing younger and older mothers (in two-parent families) there is a lack of attention to the input of the other parent despite voluminous research showing the importance of fathers' (or co-parent) contribution to family quality of life (e.g., Rohner & Veneziano, 2001). Older mothers are likely to be in partnerships with older fathers and older fathers have been shown to be more highly involved in parenting and to show more positive paternal affect than on-time fathers (Cooney, Pedersen, Indelicato, & Palkovitz, 1993). Older mothers have also been found to share more parenting tasks and rely significantly more on their partner during early infancy than do younger (on-time) mothers (Bornstein et al., 2006). These results suggest that some benefits found in older mother families could be attributed to their partner and to relational characteristics rather than to benefits arising from older age. Results in fertile and infertile populations make clear that maternal age effects need to be considered in light of partner and relationship variables. However, it should be noted that these effects may vary between populations as in a recent study with families using ART to conceive marital quality during pregnancy was poorer in families with an older mother (38 years) than in families with relatively younger (35 years) mothers (McMahon et al., 2007).

Numerous factors have been proposed to account for delayed parenthood with a common belief that such delay is not random but associated with person factors (Bewley et al., 2005) that may influence later family environment and parent wellbeing. Indeed, within-study comparisons do indicate that older mothers are more autonomous and less orientated toward parenthood than younger (on-time) mothers. Specifically, older mothers are more likely to have completed a university education, to have a high status employment (Berryman & Windridge, 1997; Robinson et al., 1987), be satisfied with their work and perceive it to be important (Berryman & Windridge, 1997), and, in terms of personality, to be more resilient, hardy (McMahon et al., 2007; Robinson et al., 1987), autonomous, and less dependent on others (Robinson et al., 1987). In contrast, younger mothers tend to have more traditional attitudes toward the role of women in society, identify more with motherhood (McMahon et al., 2007; Robinson et al., 1987) and be less rejecting of the negative aspects of care-giving than older mothers (McMahon et al., 2007). These person factors could lead younger and older mothers to provide different kinds of environments for their children (e.g., warmth, hostility) and/or to differences in maternal wellbeing (e.g., anxiety, depression). Child outcomes therefore need to be examined in relation to a wider context that includes family environment and parent wellbeing.

With this review in mind the aim of the present study was to compare families of younger and older mothers on a range of child, family and parental variables to ascertain maternal age associations and the factors that covary with these associations. We presented results with age as a continuous variable (correlation) and age as a categorical variable (analysis of variance). In the absence of consensus or justification for age cut-offs we derived the age groups for the present study on the basis of life course (“timing of events” theory) and reproductive maturity. According to Rossi (1980) parenting complications may arise from having a child off-time relative to the typical life course, and in the present study we defined typical as the national mean age at first birth in the UK (i.e., 30–31 years, see ONS, 2007) and used this mean age as the boundary for the on-time or younger motherhood group (hereafter called “younger” age group). The childbearing period in the reproductive life cycle is generally defined as between the ages of 15 and 44 years in prevalence studies (Boivin, Bunting, Collins, & Nygren, 2007) but in reality fertility is significantly reduced from 38 years onward (Collins & Crosignani, 2005). Indeed the risk of permanent childlessness is two to three times higher in women between the ages of 39 and 44 compared to those less than 30 years of age (Menken, Truddel, & Larsen, 1986). The age of 38 therefore represents a genuine biological marker of reduced expectation for fertility (Collins & Crosignani, 2005). Accordingly in the present study we defined the older mother as being 38 years or older at the time of first birth (hereafter called “older” age group). In order to ascertain whether differences between these two age groups represented linear associations with outcome variables we also compared these two groups to women who had conceived in between these ages (i.e., >31 and <38 years; hereafter called “middle” age group). All children were first-born and now aged between four and eleven years so that younger, middle and older mothers were currently about 36, 41 and 46 years of age (respectively) when reporting on their children. In order to control for previous reproductive difficulties all women in the sample had conceived the target child with ART and data about gestational and labour experiences were collected. Both mothers and fathers provided data on their own and their child's wellbeing, as well as on family environment. We therefore examined age, family environment and parental wellbeing and their association to child outcomes.

## Methods

### Participants and procedure

Couples who had a live birth between 1994 and 2002 (children aged 4–11 years) following successful fertility treatment were recruited from 20 UK clinics. All initial contact was made through clinic staff. Data were collected through postal questionnaires and mothers and fathers were asked to complete questionnaires independently. Questionnaire data from 818 families were available but only data from families where the mother had completed the questionnaire and the child was first-born (*N* = 642) were retained for analysis. 48% of families contacted returned a completed questionnaire but this is likely to be an underestimate of true response rate because some families had moved since the birth of their child 6–9 years ago and would therefore have not received the questionnaire. Of women who completed the questionnaire, (80%) also agreed to have their antenatal records reviewed.

The child who was the target of participants' responses to the child measures was first-born and conceived using ART (e.g., in vitro fertilisation, intracytoplasmic sperm injection, insemination with donor sperm). The final mother sample consisted of 642 women who were categorised into the younger (≤31 years: *n* = 158), middle (>31 years and <38 years, *n* = 311) or older (≥38 years: *n* = 173) mother age groups according to maternal age at the time of delivery. The sample also included 109, 210 and 120 spouses of the women in the younger, middle and older mother age groups, respectively. The research received ethical review and approval from the Multicentre Research Ethics Committee (Wales).

Table 1 shows age characteristics of the sample. As expected, maternal age parameters (current age, age at delivery) were significantly different among the younger, middle and older mother age groups. Partner age also differed significantly among groups (see Table 1). At the time of questionnaire completion, women in the older mother age group had been married longer and were more likely to have completed a university degree than the middle and younger groups who also differed significantly from each other on these variables. The older and middle age groups also had a significantly higher family income (>£50,000) than their younger counterparts. The average age of the target child was not different between groups, and children were on average almost seven years of age.

### Materials

The present programme of research was designed to investigate the origins of developmental disorders and the questionnaire it uses contains numerous questions on prenatal and perinatal history, family environment, child and parent behaviour and mental health. Only the items relevant to the present study are described in detail in this section but see Thapar et al. (2007) and Rice et al. (2006) for a more detailed description of overall study aims and materials.

Mothers reported on pregnancy (e.g., bleeding, high blood sugar) and obstetric complications (e.g., caesarean, ventouse delivery) using an adapted version of the Lewis, Owen, and Murray (1989) scale. Agreement between maternal report and medical records was very good for the majority of outcomes in this sample (i.e., *κ* > .80 see Rice et al., 2006). Demographic information on maternal education, family income, number of years married (or cohabitation) was also reported.

Family environment was assessed in three ways. First, mothers completed the Children's Life Events checklist and indicated which of 35 life events (e.g., being bullied, moving home) the child had recently experienced, with higher scores indicating more disruption (Johnson & McCutcheon, 1980). Second, mothers and fathers completed the Iowa Family Interaction Rating Scales (IFIRS or simply Family Interaction: Melby & Conger, 1997) and Parent–Child Interaction Scale (PCI or simply Parent–Child Interaction: Harold & Honess, 1998). The IFIRS involves rating the warmth (e.g., love, affection: 6-items) the individual expresses toward his/her partner (self-to-partner) and child (self-to-child) as well as the warmth they perceive their partner to express toward them (perceived partner-to-self). The scores for self-to-partner and perceived partner-to-self warmth were averaged in the present study to create a couple warmth score. The same ratings were obtained for hostility (e.g., criticism, anger: 4-items). The Cronbach coefficient alpha for these measures ranged from .84 to .93. The Parent–Child Interaction involved rating the extent to which interactions with the target child were positive (e.g., talk to my child in warm and affectionate way) (*α* = .84 and *α* = .78, for mothers and fathers, respectively).

Parental emotional wellbeing was assessed using several scales. Mothers and fathers completed the 14-item Hospital Anxiety and Depression Scale (HADS: Zigmond & Snaith, 1983) to derive depression (e.g., lost interest in my appearance) and anxiety (e.g., feel tense and wound up) symptom scores. The HADS has excellent psychometric properties (Bjelland, Dahl, Haug, & Neckelmann, 2002). Stress-related physical symptoms were assessed using the somatisation (e.g., chest pain, muscle tension in past three months) subscale of the Symptom Checklist (SCL-90-R) (Derogatis & Cleary, 1977). Alpha coefficient *α* = .74 and *α* = .83 for these measures.

Parents completed several measures of child wellbeing. The Strengths and Difficulties Questionnaire (SDQ or simply Strengths–Difficulties: Goodman, 1997) includes subscales assessing conduct problems (e.g., often has temper tantrums), peer problems (e.g., [not] generally liked by others) and prosocial behaviour (e.g., considerate of other people's feelings). Cronbach alpha ranged from .50 to .68 for these scales and though low was in line with research using this measure (Goodman, 2001). The short Mood and Feelings Questionnaire (MFQ or simply Child Mood: Angold et al., 1995) is an assessment of child depressive symptoms (e.g., cries a lot) that has been shown to have high internal consistency and to correlate highly with more extensive evaluations of children's depression. Anxiety was also assessed (e.g., fearful and anxious) using the symptoms for generalised anxiety in the *Diagnostic and statistical manual of mental disorders* (DSM IV, revised: American Psychiatric Association, 1994). Cronbach alpha ranged from .70 to .84 for these measures. Child health was assessed using the 7-item physical problems (e.g., stomach-ache and cramps, headaches, *α* = .62 and .63) subscale of the Child Behaviour Checklist (CBCL, Achenbach, 1991) and the “serious illness or injury to this child” from the Children's Life Events checklist. For all items higher scores imply more of the attribute (i.e., depression, physical symptoms, positive interactions).

### Statistical analyses

Distributions were examined for normality, linearity and missing data. Missing data for demographic, medical, reproductive characteristics and life events variables were not substituted, but missing data (<2%) on psychological variables were substituted with the mean of the relevant age group. Parametric (univariate and multivariate analysis of variance and covariance) and nonparametric (chi-square) analyses were used for group comparisons, as appropriate. For psychological variables multivariate analysis of variance was used to control for Type I error inflation. Analysis of covariance (univariate, multivariate) was used to examine age associations on parent (i.e., HADS-Anxiety, HADS-Depression, SCL-90-R Stress Symptoms) and child (i.e., Strengths–Difficulties, Mood, DEAM-IV-R, CBCL-Physical Symptoms) wellbeing controlling for other discriminating and explanatory variables (specific covariates indicated in Results section). Significance was defined as *p* < .05. Because of differing sample size, analyses were carried out separately for mothers (*n* = 642) and fathers (*n* = 439). Finally, 9.7% (62) of children were not living with both parents due to separation, divorce, or other life changes and these families were excluded from analyses using couple variables.

## Results

### Fertility treatment, gestational, labour and perinatal outcomes

Table 2 shows pregnancy-related characteristics. Women in the older maternal age group were significantly more likely to have used donated gametes to conceive (i.e., oocyte or embryo donation) than the middle or younger mother age groups, who were more likely to have used their own oocytes (homologous IVF, sperm donation). Women in the older and middle age groups were more likely to have had bleeding during the pregnancy, a caesarean delivery and to have breastfed compared to women in the younger age group. In contrast the middle and younger groups were more likely to have had a multiple birth than women in the older group. Mothers in the younger maternal age group were marginally more likely to smoke than the older and middle groups but this difference was not significant.

### Family environment

The age groups did not differ on the number of child life events experienced by the child, *F*(2, 616) = 2.88, *p* = .057, or in how positively fathers, *F*(2, 438) = .47, *p* = .62, rated interactions with their child. Zero-order correlations between maternal age at delivery and child life events, *r*(617) = .029, *p* = .47, and paternal ratings of time spent with child, *r*(439) = −.047, *p* = .33, were not significant. The correlation between age at delivery and maternal ratings showed that older mothers rated interactions with their child as more positive, *r*(640) = .083, *p* = .037, but the between group difference was not significant, *F*(2, 639) = 1.34, *p* = .26.

**Fig. 1:**
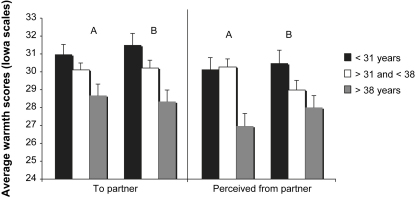
Current average warmth scores (±SEM) toward partner and perceived from partner on the Iowa Family Interaction Scale according to maternal age at delivery. For each panel, A = mother rating and B = father rating.

The multivariate analysis for family environment was significant for expressed warmth in mothers (Pillais = .04: *F*(6, 1152) = 4.37, *p* < .0001) and fathers (Pillais = .04; *F*(6, 822)=2.46, *p* = .023). There was no significant difference in maternal, *F*(2, 577) = .178, *p* = .84, or paternal, *F*(2, 412)=2.87, *p* = .058, expressions of warmth toward their child; however, couples in the older age group showed significant differences in couple expressed warmth (e.g., expression of affection, support, appreciation) (see Fig. 1). Specifically, women in the older age group reported expressing significantly less warmth toward their partner than women in the younger mother age group (Tukey, *p* < .013) and perceived receiving significantly less warmth from their partners than did women in the younger (Tukey, *p* < .001) and middle (Tukey, *p* < .001) age groups. Fathers in the older mother age group expressed significantly less warmth toward their partner than fathers in the younger (Tukey, *p* < .001) and middle mother age group (Tukey, *p* = .017) and perceived their partner to be less warm toward them than fathers in the younger mother age group (Tukey, *p* = .019). The difference between age groups in the level of expressed hostility (e.g., being critical, angry, argumentative) was not significantly different according to mother (*F*(577) = 1.02, *p* = .410) or father ratings (*F*(577) = .71, *p* = .644). Zero-order correlations between age at delivery and warmth and hostility variables were not significant except for maternal ratings of warmth toward, *r*(578) = −.129, *p* = .002, and perceived from father, *r*(578) = −.169, *p* < .0001; paternal ratings of warmth toward, *r*(417) = −.185, *p* < .0001, and perceived from mother, *r*(418) = −.120, *p* = .017, as well as paternal ratings of warmth toward the child, *r*(424) = −.131, *p* = .007. The significant correlations showed less warmth in older mother families.

**Fig. 2:**
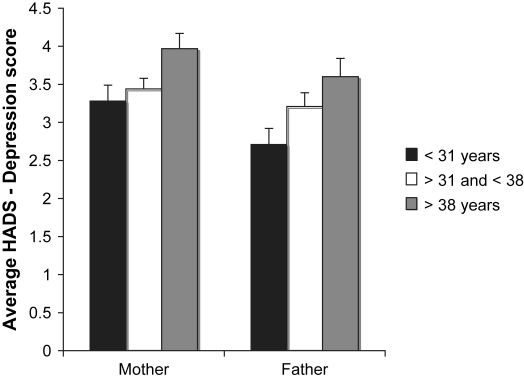
Current HADS-Depression symptom scores (±SEM) for mothers and fathers according to maternal age at delivery.

The multivariate analysis of variance on parent wellbeing was significant for mothers (Pillais = .03; *F*(8, 1274) = 2.01, *p* = .04) but not for fathers (Pillais = .03; *F*(8, 868) = 1.61, *p* = .12). The *F*-tests for maternal physical stress, *F*(2, 639) = 1.02, *p* = .36, and paternal physical stress, *F*(2, 436) = .72, *p* = .49, and maternal anxiety, *F*(2, 639) = .74, *p* = .47, and paternal anxiety, *F*(2, 436) = .10, *p* = .91,were not significant. However, Fig. 2 shows that both mothers, *F*(2, 639) = 3.51, *p* = .03, and fathers, *F*(2, 436) = 3.58, *p* = .03, from the older mother age groups reported significantly more depressive symptoms than their counterparts in the younger group (Tukey, *p* < .039, *p* = .021, respectively), but not compared to the middle age group (Tukey, *p* = .072, *p* = .359, respectively). Zero-order correlations between maternal age at delivery and parent wellbeing were not significant except for the correlations between age and mother depression, *r*(642) = .115, *p* = .003, and age and father depression, *r*(441) = .104, *p* = .03.

Analysis of covariance (ANCOVA) was used to examine which factors might account for the significant association between age and symptoms of depression in parents. For mothers, the association remained significant after controlling for income and maternal education, *F*(2, 637) = 3.83, *p* = .02, but became non-significant when controlling for use of donated [female] gametes, *F*(2, 638) = 1.91, *p* = .15, or relationship variables (i.e., years with partner and mother-rated couple expressed warmth) *F*(2, 583) = .28, *p* = .76. For fathers, the association remained significant when controlling for income and education, *F*(2, 434) = 3.68, *p* = .03, or use of donated [male] gametes, *F*(2, 435) = 3.58, *p* = .03, but became non-significant after controlling for relationship variables (i.e., years with partner and father-rated couple expressed warmth) *F*(2, 417) = .26, *p* = .77.

### Child wellbeing

Mother and father ratings of child wellbeing are shown in Table 3. The scores on subscales of the Strengths–Difficulties, Depressive Mood, Anxiety, and Physical Symptoms were similar among age groups for both mothers and fathers. Multivariate analyses comparing age groups on these child outcome variables were not significant for mother ratings (Pillais = .03; *F*(12, 1270) = 1.45, *p* = .138) or father ratings (Pillais = .04; *F*(12,868) = 1.49, *p* = .121). These analyses were repeated using multivariate analysis of covariance controlling for the variables that showed age-group differences (i.e., number of siblings, education, income, years together, use of donated gametes, use of caesarean, breast feeding, couple warmth and parental depression) and these were not significant for mothers (Pillais = .03; *F*(12, 1090) = 1.33, *p* = .195) or fathers (Pillais = .04; *F*(12, 782) = 1.36, *p* = .178). In addition, there was no difference between groups in the frequency of serious injury or illness in the target child as recorded in the child life events checklist, *χ*^2^(2) = .783, *p* = .68.

## Discussion

Our results show that parenting later in the reproductive life cycle was not associated with poorer child outcomes in early and middle childhood on the emotional and behavioural problems that we measured with no evidence of psychosocial advantage or disadvantage for children in any maternal age group. We further conclude that the parenting context is different in older mother families (less expressed warmth between spouses, more depressive symptoms in mothers and fathers) but that this difference is more likely due to age and time-dependent biological and relationship changes than to parenting at an older age.

The differences observed between younger and older mothers on demographic and medical variables reflect the potential advantages and disadvantages of delayed childbearing. On the one hand older mothers were better educated, had higher family incomes and took fewer risks during the perinatal period (less likely to smoke, more likely to breastfeed). The pursuit of greater economic stability through career development has previously been reported (Berryman & Windridge, 1997; Robinson et al., 1987) and proposed to be a main cause of delayed parenting (Bewley et al., 2005) but one which can ultimately benefit the family (Hofferth, 1987) as would a more cautious approach to pregnancy and early infancy. On the other hand, our results show that because of reduced reproductive capacity, delay also meant that older mothers were more likely to need the donated oocytes of a younger woman to conceive and to experience more negative gestational and labour events (i.e., vaginal bleeding and caesarean delivery) and that such costs were linearly related to age. These findings are consistent with those of other medical studies (for review see Bewley et al., 2005) and confirm that couples wanting to become parents need to consider educational, employment and economic goals in the wider context of increased physical risks to the mother and offspring from delaying conception.

Our results demonstrate for the first time that parenthood via assisted conception later in the reproductive life cycle is not associated with a negative impact on child wellbeing in terms of the emotional and behavioural factors we measured in early to middle childhood. Past findings regarding better parenting practices in older mothers during infancy (<1 year) (e.g., Bornstein et al., 2006) were not replicated in mothers with children in our age range of 4–11 years. Outcome analyses showed that children in all age groups were growing up in warm and caring environments as reported by their parents. Parents in all groups enjoyed interactions with their child, for example, praised their child, felt proud of the child's achievements and considered their child's activities to be important. Parents reported that children experienced few stressful life events or hostility (arguments, criticism, anger) from either parent. Children in younger, middle and older parent groups were similarly perceived by their parents in relation to strengths and difficulties, mood (anxiety and depressive symptoms) and experience of stress-related physical symptoms (e.g., aches and pains, stomach upsets) and child scores were comparable to norms (Shelton et al., in press). Finally, the similarity across children remained even when examined in relation to other factors that differentiated the age groups (e.g., income, education, gestational factors, parental depression). There is a large literature showing that parental reports of child mental health are valid in that they predict functional outcomes in adult life (e.g., Ferdinand, Stijnen, Verhulst, & van der Reijden, 1999) and correlate with reports made by other informants (e.g. teachers, examiners, observers, see Arseneault et al., 2003) nevertheless our claim that children do not appear to have benefited or suffered thus far from having an older mother would be strengthened by using other informers and methods (e.g., observation as used in Bornstein et al., 2006).

The costs of delayed parenting may come about more from age and time-dependent life changes (that all experience) than to the particular act of having children at an older age. Age can be a proxy for underlying sociological, psychological and biological changes (Cooney et al., 1993) and these may change the parenting context in older mother families. Our results point to two such changes, one relational the other biological, and our secondary analyses suggest that these changes likely explain the association between age and parental depression. One change is indicated by the longer partnership (by 3–5 years) and lower expressed warmth in couples of older mother families. Past research shows a more or less continuous decline in expressed warmth, love and affection over the course of a marriage (Van Laningham, Johnson, & Amato, 2001) and our covariate analysis indicated that the lower parental wellbeing in older mother families seemed secondary to this time-related change in the couple relationship rather than to the act of being a parent at an older age.

Similarly, the parenting context may be affected by age-related biological changes. The use of donated oocytes also accounted for maternal (but not paternal) depression and this finding may point to a biological explanation for age-group differences in maternal depression. The biological event that causes the need to use donated oocytes, i.e., the onset of menopause, could also be implicated in producing higher depressive symptoms in older women (Pearlstein, 1995). We did not assess menopausal symptoms in our sample but 30% of older women were in the peri-menopause (>48 years old) with 10% post-menopausal (>51 years of age) and therefore potentially experiencing the symptoms associated with this biological event, that is, greater vasomotor symptoms, sleep disturbance and sexual dysfunction (see Nelson et al., 2005 for systematic review). Such symptoms could account for age-related associations in maternal depression as well as reduced warmth in couples of older mother families.

Together these findings suggest that the benefits of having children within the “typical life course” may come not only from advantages caused by being developmentally on-time with peers (Rossi, 1980) but also from the benefits of not having children at a time that coincides with other age or time-dependent biological or relational changes that alter the parenting context and make parenting more difficult for the couple. This possibility could better be explored in designs that took into account the biological and social context of parenting.

The strength of the present investigation was that age groups were formed according to the population mean age at first birth (ONS, 2007) and the biological marker of reproductive decline (Collins & Crosignani, 2005), which ensured a meaningful difference between groups for age at first birth: about a decade difference in mean age and 20 year difference in the age of the oldest woman in the younger group (31 years) and her counterpart in the older mother age group (50 years). We presented age associations using this categorical age variable and as a continuous variable and results showed convergence using both methods. The sample of older mothers was about twice the size of previous cohort controlled studies (Berryman & Windridge, 1997; McMahon et al., 2007; Robinson et al., 1987) with sufficient power to detect medium to large effect sizes. Our participation rate of 48% was satisfactory and in keeping with that of UK samples in other family surveys (see Barnes et al., 2004, Table 1). Furthermore, the pregnancy was planned in all participants, the target child was the first-born for all women, and all couples used ART to conceive, thus avoiding confounding effects attributable to reproductive history. In the case of medical variables women's self-report recall of gestational and labour complications corresponded to a high degree with their medical records (see Rice et al., 2006 for details). Ratings of family environment and child wellbeing were mainly based on sound psychometric instruments and were provided by mothers and in about 70% of cases fathers as well. Together, these strengths increase confidence that the associations observed in the present study are reliable and valid.

There were some limitations. As our study was, by necessity, a cross-sectional design the direction of causality between maternal age and discriminating variables cannot be conclusively determined. In particular, we do not know whether educational attainment and income is a cause of delayed parenthood (as proposed previously) or a by-product of taking a long time to conceive. We also do not know whether the couple profile presented by older mother groups (higher symptoms of parental depression, lower couple warmth) was present at the start of fertility treatment and caused delay in parenting or whether it was a consequence of reproducing late in the female life cycle, as we have argued. Only prospective investigations can lead to definitive conclusions. Although parental reports were as valid indicators of the outcomes as could be obtained considering ethical issues (sensitivity in an ART sample) and practical issues (self-reports in younger children are problematic), sole reliance on parental reports for all psychosocial outcomes may introduce a social desirability bias not present in studies using external informants (e.g., teacher ratings, observational methods, e.g., Bornstein et al., 2006). Further, one of the child questionnaires showed low reliability (though values were consistent with author reports for this measure, see Goodman, 2001). Replication using other informants and other measures would therefore be important to strengthen the claims made in the present report.

Finally, to whom these results can be generalised might be questioned because all couples had conceived with fertility treatment. Our age results were consistent with past work on naturally conceiving couples, for example, associations between age and education, gestational and labour events, and between warmth and partnership duration. Although it is possible that ART and naturally conceiving couples differ on mean values for the constructs there is no evidence to support a proposal that the nature of relationships between these constructs (e.g., association between warmth and partnership duration) would differ according to method of conception. Consequently, the age associations observed in this study add to existing work on older motherhood in naturally conceiving couples too but replication in a naturally conceiving population would be important.

The results of this study show that there can be some advantages for families in mothers conceiving after the age of 38 (education, income), but clearly balancing these against costs (gestational problems, onset of other biological and relational changes) should be an important issue in couple decision-making about when to become parents. Importantly though, parental reports suggest that children in early and middle childhood do not appear to be adversely affected by the older age of their mothers in terms of the emotional and behavioural problems examined in the present study.

## Figures and Tables

**Table 1:** Zero-order correlation, and mean (SD) or percent (*n*) for demographic characteristics of the sample (*N* = 642) according to maternal age at delivery.

Variable	Zero-order correlation with maternal age at delivery	Younger	Middle	Older	
		<31 years	>31 and <38	>38 years	
		(*n* = 158)	(*n* = 311)	(*n* = 173)	
		Mean (SD)	Mean (SD)	Mean (SD)	(dfs), *t*-value
Current maternal age	–	36.03 (2.10)^a^	41.03 (1.99)^b^	46.91 (3.19)^c^	(639), 855.08***
Current paternal age	.509***	33.34 (4.74)^a^	37.67 (5.22)^b^	41.92 (5.88)^c^	(632), 88.25***

Maternal age at birth	–	29.28 (1.62)^a^	34.48 (1.58)^b^	40.47 (2.84)^c^	(639), 1289.17***
Range (years)		21–31	32–37	38–50	
Paternal age at birth	.516***	40.11 (5.06)^a^	44.23 (5.37)^b^	48.22 (6.11)^c^	(632), 106.77***

No. years together	.345***	14.12 (3.34)^a^	15.77 (4.04)^b^	18.56 (5.62)^c^	(583), 40.61***

Child's age (years)	–	6.81 (1.23)	6.76 (1.23)	6.62 (1.31)	(639), 1.08
Mean number of siblings	−.170***	.94 (1.0)^a^	.84 (.9)^a^	.51 (.8)^b^	(637), 9.91***

		% (*n*)	% (*n*)	% (*n*)	*χ*^2^
% Children not living with both parents	.057	8.2 (13)	10.0 (31)	10.4 (18)	(2), .515
University degree	.218***	16.5 (26)^a^	30.2 (94)^b^	42.2 (73)^c^	(2), 26.03***

Family income					
<20,000	−.021	17.7 (28)^a^	15.8 (49)	15.0 (26)^b^	(2), .482
20,000–50,000	−.073	61.4 (97)	49.8 (155)	52.0 (90)	(2), 5.77
>50,000	.110**	17.7 (28)^a^	31.5 (98)^b^	30.6 (53)^b^	(2), 10.80**

*Note*. Sample size varies due to missing data, dfs provided. For number (no.) years together, *n* = 62 children were not living with both parents. *** *p* < .001; ** *p* < .01. Means with different superscripts differ significantly.

**Table 2:** Zero-order correlations and percents (*n*) for fertility treatment, gestational, labour and postnatal characteristics according to maternal age at birth.

Variable (% yes)	Zero-order correlation with maternal age at delivery	Younger	Middle	Older	*χ*2(2) value
		<31 years	>31 and <38	>38 years	
		(*n* = 158)	(*n* = 311)	(*n* = 173)	
*Type of IVF conception*		% (*n*)	% (*n*)	% (*n*)	
Homologous	−.238***	57.6 (91)^a^	62.1 (193)^a^	30.1 (121)^b^	47.96***
Sperm donation	−.124**	27.8 (44)^a^	22.8 (71)^a^	17.3 (30)^b^	5.23^t^
Oocyte donation	.296***	12.7 (20)^a^	12.5 (39)^a^	39.3 (68)^b^	56.89***
Embryo donation	.247***	1.3 (2)^a^	1.6 (5)^a^	10.4(18)^b^	26.86***

*Gestational factors*					
Smoking	−.088*	8.4 (13/155)^a^	3.9 (12/310)^b^	3.5 (6/173)^b^	5.52^t^
Alcohol^a^	.023	22.9 (36/157)	24.8 (77/310)	24.9 (43/173)	.24
Prescription medicine^b^	−.055	31.2 (49/157)	26.8 (83/310)	27.9 (48/172)	1.02
High blood sugar^c^	−.016	8.9 (14/157)	7.0 (21/302)	7.2 (12/167)	.61
Vaginal bleeding	.159***	21.5 (34/158)^a^	31.5 (97/308)^b^	38.4 (66/172)^b^	11.07**

*Labour, birth & postnatal factors*					
Caesarean	.132***	38.6 (61/158)^a^	43.4 (135/311)^b^	52.0 (90/173)^b^	6.33*
Ventouse delivery	.012	24.1 (38/158)	28.9 (90/311)	25.4 (44/173)	1.50
Multiple birth	−.060	22.2 (35/158)^a^	21.9 (68/311)^a^	13.3 (23/173)^b^	6.02*
Infant breastfed	.116**	66.2 (104/157)^a^	77.5 (241/311)^b^	80.9 (140/173)^b^	10.73**

*Note*. Missing data were not substituted for medical variables therefore parentheses for gestational factors and labour, birth and postnatal factors indicate *n* for category/number of respondents to item. Frequencies with different superscripts are significantly different. ^t^*p* < .10; * *p* < .05, ** *p* < .01, *** *p* < .001.

**Table 3:** Zero-order correlations and means (SD) on child wellbeing ratings by mothers (M) and fathers (F) according to maternal age at birth.

Variable		Zero-order correlations with age at delivery	Younger	Middle	Older
			<31 years	>31 and <38	>38 years
			(*n* = 158)	(*N* = 311)	(*n* = 173)
*SDQ*					
Conduct problems	M	.028	1.38 (1.3)	1.40 (1.4)	1.50 (1.4)
	F	.058	1.48 (1.4)	1.42 (1.3)	1.64 (1.5)
Peer problems	M	.060	1.25 (1.4)	1.11 (1.5)	1.55 (1.7)
	F	.027	1.38 (1.4)	1.25 (1.4)	1.51 (1.5)
Prosocial behaviour	M	−.027	8.48 (1.6)	8.39 (1.6)	8.36 (1.8)
	F	−.100*	8.55 (1.6)	8.30 (1.6)	8.16 (1.7)

MFQ depression	M	.024	2.61 (2.6)	2.75 (2.7)	2.98 (2.8)
	F	.013	2.48 (3.1)	2.83 (2.9)	2.54 (2.5)
DSM IV-R anxiety	M	−.034	2.20 (2.2)	2.40 (2.2)	2.23 (2.2)
	F	−.022	1.97 (2.4)	2.32 (2.3)	1.94 (1.9)
CBCL: physical symptoms	M	−.056	1.81 (1.9)	1.63 (1.71)	1.66 (1.60)
	F	−.035	1.46 (1.6)	1.47 (1.7)	1.37 (1.6)

*Note*. M = mothers, F = Fathers; SDQ: Strengths and Difficulties Questionnaire; MFQ: Moods and Feelings Questionnaire; DMS-IV-R: Diagnostic and Statistical Manual of Mental Disorders, version IV-Revised; CBCL: Child Behavior Checklist. *p* > .05 for all tests between mother age groups., * *p* < .05.
